# Gaze Strategy in the Free Flying Zebra Finch (*Taeniopygia guttata*)

**DOI:** 10.1371/journal.pone.0003956

**Published:** 2008-12-24

**Authors:** Dennis Eckmeier, Bart R. H. Geurten, Daniel Kress, Marcel Mertes, Roland Kern, Martin Egelhaaf, Hans-Joachim Bischof

**Affiliations:** 1 Lehrstuhl für Verhaltensforschung, Universität Bielefeld, Bielefeld, Germany; 2 Lehrstuhl für Neurobiologie & Center of Excellence‚ Cognitive Interaction Technology, Universität Bielefeld, Bielefeld, Germany; University of Southern California, United States of America

## Abstract

Fast moving animals depend on cues derived from the optic flow on their retina. Optic flow from translational locomotion includes information about the three-dimensional composition of the environment, while optic flow experienced during a rotational self motion does not. Thus, a saccadic gaze strategy that segregates rotations from translational movements during locomotion will facilitate extraction of spatial information from the visual input. We analysed whether birds use such a strategy by highspeed video recording zebra finches from two directions during an obstacle avoidance task. Each frame of the recording was examined to derive position and orientation of the beak in three-dimensional space. The data show that in all flights the head orientation was shifted in a saccadic fashion and was kept straight between saccades. Therefore, birds use a gaze strategy that actively stabilizes their gaze during translation to simplify optic flow based navigation. This is the first evidence of birds actively optimizing optic flow during flight.

## Introduction

Navigating through a complex environment requires a specific set of information. It is essential to quickly get an impression of the three dimensional composition of the environment. This impression would consist of the distances between the observer and the objects in the environment, as well as among those objects. Such information may be used to anticipate the future path of movement, and to decide when to execute manoeuvres necessary to follow that path without the risk of collisions.

Several mechanisms are known to allow the estimation of distance, but doing so during fast locomotion presents special challenges. Sharp retinal images of objects or edge detection are very difficult to obtain due to motion blur. Also, using accommodation mechanisms for distance estimation would be too slow for fast navigation in difficult and unknown terrain [1]. Stereopsis is a major cue for sensing depth, but it requires binocular viewing which is well developed only in predators [2]. Further, stereopsis works also only within a limited spatial range depending on the distance between the eyes and the spatial resolution [3]. All these mechanisms are therefore not ideal for distance estimation during fast navigation, especially in rapidly flying animals such as many birds.

Optic flow fulfils the requirements for fast detection of information relevant for visually guided navigation of fast moving animals. It refers to the velocities with which environmental objects are displaced in the retinal image of the moving animal. Optic flow follows basic geometric rules that allow a moving human or animal observer to estimate its relative distance to environmental objects by analyzing these movements [4]–[6]. Detailed edge or object recognition is not necessary. It is sufficient to detect areas of different speed and direction of motion by means of optic flow.

Optic flow is produced by self motion: During straight (translational) motion, the retinal images of objects in the visual field move with different velocities according to their distances from the observer. The images of objects that are far away move slowly while those of near objects move fast. In addition, the images of approaching objects expand while images of receding objects contract. Hence an animal can estimate distances to and among objects from the optic flow experienced during translational self-motion. However, many movements have an additional rotational component due to turns of the head or the body. The optic flow generated by such rotational movement does not provide any distance information because the velocities of retinal images of differently distant objects do not differ [4]. This may complicate the processing of distance information provided by the translational optic flow component. The extraction of distance information from optic flow could thus be facilitated if its rotational component is reduced by an active gaze strategy. Schilstra and van Hateren [7]–[9] showed that blowflies separate rotational from translational motion by following a flight path with straight passages interrupted by very fast saccadic turns of the body and head instead of flying in smooth curves.

Here we investigate whether birds exhibit similar active gaze behaviour that would facilitate the use of optic flow during free flight. Many avian species move very fast in three dimensions and, therefore, may have evolved a well developed a navigational system based on optic flow. As yet, behavioural evidence that birds actually make use of optic flow during flight is rare, probably because their size and speed would require too much space. The few experiments that have been done focussed on a single task such as plummeting or landing [10]–[12].

We filmed zebra finches flying around an obstacle with two high-speed cameras, and analyzed the recorded head movements to obtain information on their gaze strategies during flight. Eye movements were neglected for methodological reasons. This protocol is justified by the findings of Gioanni et al [13], [14], who demonstrated that in the combined optokinetic and optocollic reflex, the head movements account for 80–90% of the overall gaze shift. So the birds mainly move their heads when changing gaze direction. If we could show that head turns were restricted to short periods of the flight, this would be a strong hint that the distance information needed for navigation might be obtained from optic flow cues.

For insights into natural optic flow processing in the brain, the flight behaviour of white zebra finches might be of special interest. This albinotic mutation is known to have strong anatomical and physiological changes of the central visual system leading to enhanced neuronal responses in areas ipsilateral to the stimulated eye [15]–[17]. Some of the areas with enhanced responses in the white birds feed information to nuclei of the accessory optic system that processes optic flow. Other areas involved in distance estimation like nucleus rotundus in turn receive input from the accessory optic system [18] This may lead to a less efficient flight performance. To relate possible deficits of flight behaviour to neuronal deviations may then help to identify the neuronal structures essential for the processing of optic flow.

The primary goal of the present experiments is to examine whether zebra finches use a behavioural strategy to separate the translational and the rotational component of optic flow. As stated above, only the translational component, that is optic flow induced by straight flight, contains the distance information needed for manoeuvring. Turning movements of the head, which add a rotational component of optic flow, should therefore be avoided. If turning movements are necessary as for example when flying around an obstacle, a bird should develop a strategy where turning movements and straight flight alternate instead of being intermingled. Demonstration of such a strategy would prove that optic flow is an important tool for flight path control and may be universally used throughout the animal kingdom.

## Materials and Methods

The experiments were performed with the zebra finch (*Taeniopygia guttata*), a small Australian songbird, raised and kept at the department's animal care facilities. Ten individuals were accustomed to the flight arena, 5 of them being white, the others wild type birds of the normal grey colour.

**Figure 1:**
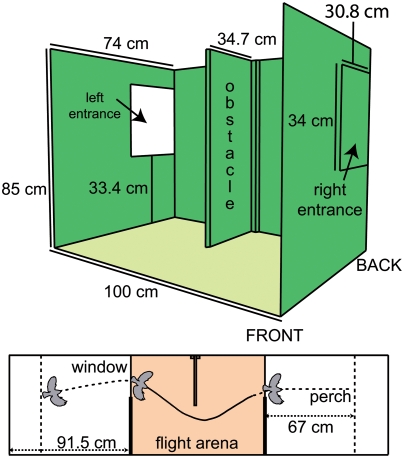
The flight arena. During experiments the bird entered the middle division through one of the entrances, avoided the obstacle by flying around it, and left through the opposite window. Roof and front were open for video recording. The walls were lined with random texture to make navigation by landmarks difficult. The floor was covered with single coloured paper to increase contrast in video recordings from above.

The flight behaviour of the bird was examined in a cage especially built for this experiment. It was 283 cm long, 85 cm high and 74 cm deep and separated by wooden walls into three compartments. The central compartment was 100 cm long, the outer ones about 90 cm (fig. 1). The birds could enter and leave the central compartment by windows in the partitions, each 31 by 34 cm, located at the rear of the cage at a height of 38 cm above the floor. The central compartment was divided by a 1.5 cm thick wall (obstacle) reaching from bottom to roof and extending from the back end into the cage by 34.7 cm. In the front wall was a window (90×36 cm). The outer compartments of the flight arena had one perch each (67 cm away from the window and 40 cm from the floor) and walls of mesh wire (1.2 cm mesh width).

The walls and the obstacle were wallpapered with green to black randomly textured paper to make edge detection more difficult as we did not want the birds to navigate by landmarks. The roof and the floor were not wallpapered. The floor was covered with plain sand-coloured paper because wild type birds were hard to recognise on the video recordings from above when textured wallpaper was used. During experiments the roof was left open for illumination and for video recording from above. On top of the open roof, a box was installed with openings for spotlights and a camera. Lights and camera were mounted on top of the box. The second camera faced through the front window of the middle part of the flight arena (shown in fig. 1), which was also left open to avoid reflections by an otherwise necessary glass window. During training the roof was covered with wire mesh and the front window was closed with a pane of glass. So the birds learned not to fly through these openings and none of them did in the experiment. Additionally, the area in front of the cage was darkened by black cloth during the experiments. This prevented the birds from leaving the cage and eliminated unwanted hints by landmarks outside the cage. The second camera was installed within this darkened area for video recordings from the front.

For training as well as during the experiments, a bird was put into one of the outer compartments and perched there. It was then forced to fly by approaching the cage or by a piece of cloth applied to a string and moved from the outside of the cage. In response to these actions, the bird then entered the central compartment through one of the windows, flew around the obstacle, left the central compartment through the other window, and perched at the other outer compartment. It was then forced to fly into the opposite direction. Flights in both directions were recorded during experiments. Training continued for four days until every bird passed the middle compartment without landing inside or touching the (transparent) glass window at the front side of the cage. Then the glass pane at the front and the mesh wire at the top of the central compartment were removed to allow video recordings. As stated above, the birds did not attempt to fly through these opening during experiments.

High speed cameras (Red Lake Motion Pro; 500 frames/s) were used for video recording. The top camera was situated 125 cm above the upper rim of the cage with a 12.5 mm objective. The front camera stood 153 cm away from the front rim of the cage. For later analysis the recordings of both cameras had to be synchronized. This was accomplished by using the Red Lake ‘Midas’ software.

**Figure 2:**
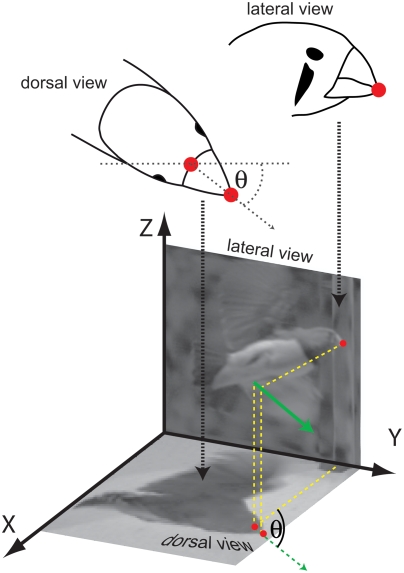
Determination of the gaze direction. In a pair of image frames taken from different directions, the base and the tip of the beak were marked as is indicated by red dots. Theta (⊖) is the angle between the X axis and the beak axis from a dorsal view or the horizontal orientation angle of the beak. The three-dimensional position of the beak tip is determined from both pictures. Dotted green arrow indicates gaze direction in dorsal view; solid green arrow is projected in three dimensions.

To reconstruct the position and orientation of the bird's head we manually marked discrete points of the bird's beak in every frame of both recordings with the help of ‘Fly Trace’ [19], a custom made software that returns pixel coordinates of marked positions in a bitmap picture. In single frames of videos taken from above, the tip and the base of the beak were marked. In videos taken from the front, providing a side view of the flight, only the beak tip was marked in each frame (fig. 2). Noise introduced by this manual tracing was accounted for by smoothing the data using a Gaussian filter. The frequencies that had to be filtered out were determined by analysing the frequency spectrum of the noise generated by ten different people, who digitized the same video sequence. The resulting pixel coordinates obtained from both views were stereo-triangulated to derive three-dimensional position and orientation of the beak as projected into the horizontal plane (fig 2).

**Figure 3:**
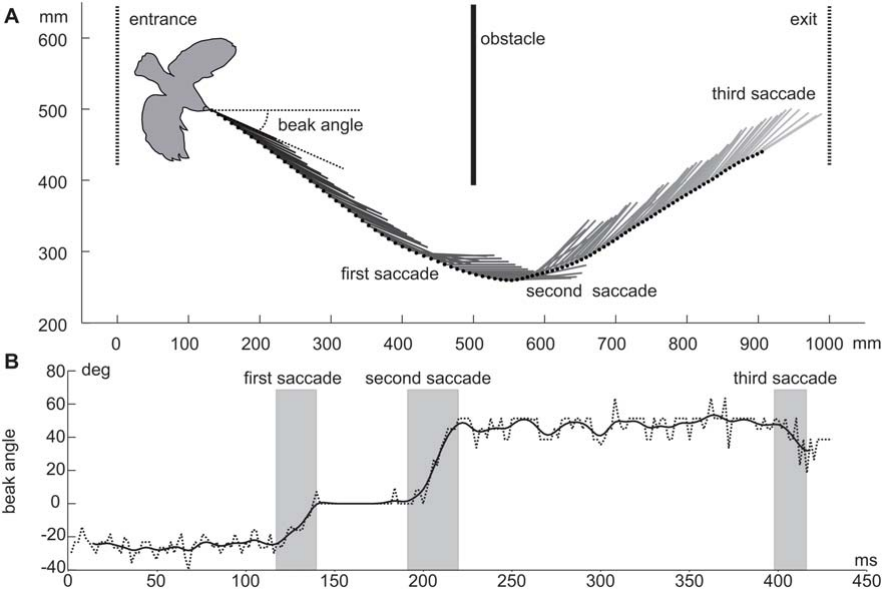
Example of a flight trajectory. A: Position and head orientation of a bird shown every 4 ms within the middle division of the flight arena. Dots indicate beak tip position; lines indicate horizontal beak orientation and, thus, gaze direction in the middle section of the flight arena. The bird contour is taken from the first frame analysed and approximately sized relative to the indicated cage dimensions. Origin of XY plane is the front left corner of the middle division. Positions of entrance, exit and obstacle are marked. B: Time-dependent beak angle relative to long axis of flight arena over time. The dotted line indicates raw data and the solid line indicates filtered data.

We calculated the beak orientation within the horizontal plane from the position data of the base and the tip of the beak to estimate gaze direction (see Discussion for the problem of “gaze direction” in birds). Eye movements could not be measured, but given that head movements account for up to 90% of a gaze shift [13], [14], the beak direction was interpreted to coincide with the gaze direction (fig 2, 3). The angular velocities of gaze changes were calculated by determining the changes in beak orientation between two frames and relating them to the video frame rate (500 frames/s).

As we wanted to reconstruct the position and orientation of the head in three dimensions, we had to obtain calibration data that allowed us to calculate the real spatial position from the pixel coordinates. This was done with the J.Y. Bouguett camera calibration toolbox for Matlab (MathWorks USA) [20]. We used 31 interpolation points that were physically defined as the tops of upright bars on a so called ‘Manhattan’ model. The bars were of different but defined heights and positions within the flight arena (removed before training and experiments). Pixel coordinates of these interpolation points were taken from single frames. We employed an optimisation routine to choose four points, which then were used to build a translational matrix. Based on this translational matrix, we also derived a rotational transformation matrix. With these matrices we were finally able to calculate the real position of the objects in the arena from the pixel coordinates derived from the recordings. The calculations were done using Matlab (Mathworks Inc.). Most scripts used here were based on scripts previously developed in our group, but adjusted or rewritten for the purposes of the current study.

To search the data for sequences of high rotational head velocities, that is saccadic gaze shifts, we defined two search parameters. First, the angular velocity had to be larger than 400°/s for at least four consecutive frames (i.e. for at least 8 ms). Second, the angular velocity had to reach a peak of at least 700°/s during such a turn.

Finally we examined the orientation of the head in the vertical plane. We randomly selected three birds of each morph and analysed the recordings of the lateral view of their flights. Only the short flight path intercepts where the birds flew parallel to the frontal border of the cage (almost perpendicular to the camera axis) allowed us to obtain the pitch angle of the head exactly enough. The raw head orientation values within each flight were normalised by subtracting the mean orientation to get a single dataset for each bird.

The original research reported herein was performed under guidelines established by the German Welfare Law.

## Results

We recorded 97 flights in a flight arena with an obstacle. Fifty of these were performed by white zebra finches, 47 by wild type birds. Due to the experimental procedure, about half of the flights (46) were from left to right comprising a left turn around the obstacle in the central compartment, the other half (51 flights) was from the right to the left with a left turn around the obstacle. Neither the colour morph nor the flight direction affected the experimental results. We therefore pooled the entire data set.

The recordings were made after the birds had been acclimatized to the cage and reliably traversed the central compartment without colliding with the obstacle or arena walls. The birds flew with a relatively high speed of 2.49±0.033 m/s, so that the central compartment was usually crossed in less than half a second. Accordingly, few wing beats were performed. The wings were opened only when a bird changed its flight direction. In between, they were flattened along the body (Video S1).

The birds had to fly into the middle compartment of the cage through an entrance facing the obstacle. Therefore, a bird entering at the left entrance first had to turn right, then perform a leftwards turn to fly around the obstacle and eventually turn right to reach the exit window. Accordingly the turns were in opposite direction when the bird entered the central cage from the right entrance. Although the birds executed two or three turns in the setup, only the turn around the obstacle was reliably recorded. The other turns occurred at the beginning or end of the flight and were often recorded incompletely or not at all. We, therefore, limited our analysis to the turn around the obstacle.

**Figure 4:**
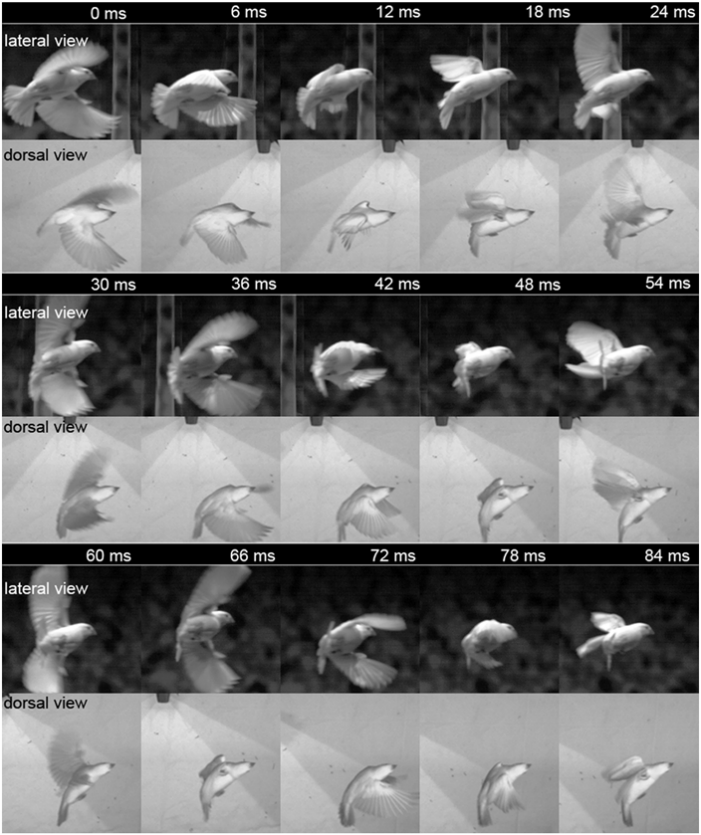
Sequence of a manoeuvring bird. As shown in this series of picture pairs, during a braking manoeuvre the head stays steady while the body turns and the tail feathers are spread.

When the bird flew around the obstacle, it decelerated and turned into the new direction of its flight path. During this manoeuvre, the wings were opened and the body turned and pushed forward relative to the head while the tail feathers were spread (fig. 4). Then the body turned into the new flight direction facilitated by wing beats. Although the body showed substantial twisting during these turns (fig 4), the head kept it's orientation in space, suggesting that the bird kept its gaze constant between turns. During breaking in turns the height of the birds decreased for a short period; nonetheless, up and down movements were small. The differences between the highest and lowest point ranged between 2 and 30 cm and on average across flights, amounted to 10.49±5.65 cm.

Fig. 3A shows an example of a flight from the left to the right with a left turn around the obstacle. The dots along the flight path depict the position of the beak tip (used as a marker for the position of the head) every second frame. The direction of the short lines represents the gaze direction. Although the movement of the head is a smooth curve, the gaze direction remains relatively constant over quite long periods of time. Such phases of relatively constant gaze are interrupted by short and fast changes of head direction, i.e., by head saccades. Hence, the head is not turned continuously, but changes its orientation in short distinct phases. This is demonstrated in figure 3B.

**Figure 5:**
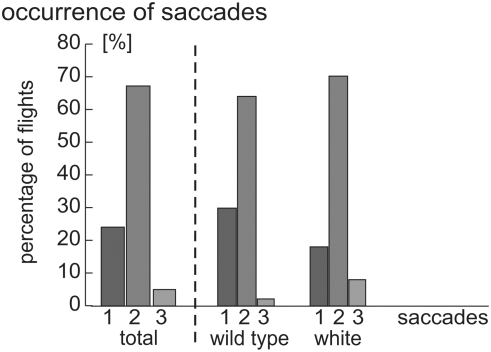
Percentage of saccade counts per flight. The figure is subdivided into 1 to 3 saccades for each morph and summed for all birds (n = 174).

To define criteria for a computer-based identification of saccades we examined results such as presented in fig 3B. Earlier results that showed that the zebra finch does not respond optokinetically to rotational flowfields faster than 349 (±67)°/s further supports our decision [21]. For this reason we defined saccades as being the periods in which the absolute value of the angular velocity of the beak was above 400°/s for at least 8 ms, and with the peak maximum being at least 700°/s. By applying these criteria, we found at least one saccade in every flight (fig. 5) except one. Two saccades were observed for 66 (68%) of the flights. Only one saccade was found in 22 (23%) flights. Few flights showed three saccades (7; 7%), and one (1%) flight even comprised four saccades. There was one flight that did not reveal any saccade matching the search parameters, but the video showed smaller saccadic turns also for this flight. Despite this limitation, the parameters used for saccade detection probably are a good compromise between missing relevant saccades and the detection of spurious events that do not represent saccades. The saccade number distribution in white and wild type zebra finches was very similar (fig. 5).

**Figure 6:**
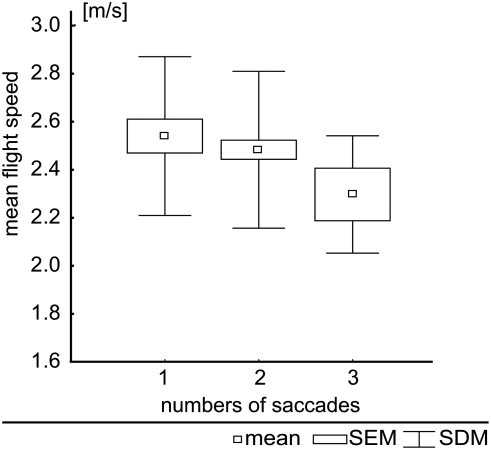
Mean flight speed. Mean flight speed for flights with 1 to 3 saccades (n = 95). The small squares indicate means, boxes indicate the standard error of the mean, and whiskers indicate standard deviations.

The number of saccades made during a flight depends on the speed of the bird along the flight trajectory. It slightly decreased with increasing speed (fig. 6), although this trend is not statistically significant for our data base (Kruskal-Wallis-Anova: H = 1.236, N = 97, p = 0.8721).

**Figure 7:**
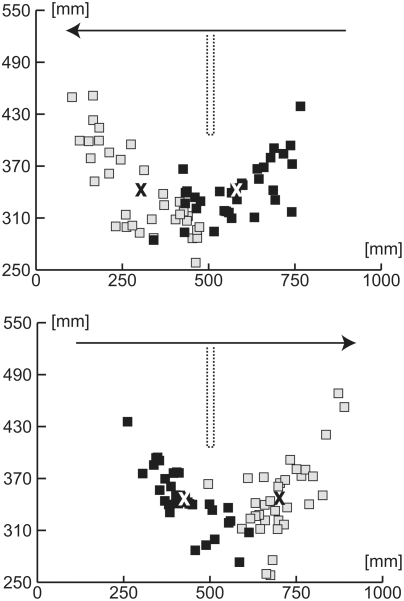
Saccade positions of flights with two saccades. The axes correspond to long and transverse axes of the middle division of the flight arena. The dotted line indicates the position of the obstacle. The arrow indicates the flight direction while squares indicate beak position at the beginning of the saccade (black squares: first saccade; white squares: second saccade). X indicates the position of the mean value (white X: first saccade; black X: second saccade).

The spatial distribution of the first and second saccades (black squares and white squares, respectively) is rather broad, occurring almost at any location along the analysed section of the flight trajectories (fig. 7). However, the distribution of the first and the second saccades is not symmetrical with respect to the obstacle (see the position of the crosses which depict the mean of the first and the second saccades, respectively). In both flight directions, the first saccade is closer to the obstacle than is the second one.

Up to now we have shown that birds are using a saccadic gaze strategy in our flight arena: they are alternating during flights on a curved path between times where they keep the head orientation relatively constant followed by saccadic head movements.

Figure 3 may cast doubts on the intersaccadic constancy of the head direction. The orientation of the beak fluctuates slightly even after filtering as long as it is not 0°. When we recognized this, we re-examined our videos and found intersaccadic intervals in 18 flights during which the beak was oriented around 0° (one of them depicted in fig. 3). In any case the fluctuations of beak orientation within these flight sections were almost negligible. A beak orientation of 0° indicates that the beak was oriented parallel to the X axis in the above view. We therefore think it likely that single pixels can be marked more exactly for a 0° beak orientation than is true for other angles and that fluctuations of the beak orientation during other intersaccadic intervals may be an artifact. Hence, we may conclude that the gaze direction is well stabilised during the intersaccadic intervals.

The mean angular velocity of saccades (n = 178) was about 1082.89±22.43°/s, the fastest saccade reaching 2154°/s. Average angular speed of intersaccadic intervals (n = 82) was about 114.75±7.86°/s. So between saccades head rotational movements in the horizontal plane were comparably slow (see also the previous paragraph). The mean duration of saccades (defined by velocities above 400°/s) was 15.6±0.4 ms, while intersaccadic intervals lasted 91.9±3.93 ms. This means that when seen from above, the head was held in a constant direction for 83% of the flight around an obstacle.

Usually, when examining rotations, changes and velocities are presented with algebraic signs to indicate direction. Here we pooled saccades of different direction and, therefore, used absolute values of the data. This could be done because all flights from one direction (left or right turn) only produced saccades of the same direction (see figure 3).

**Figure 8:**
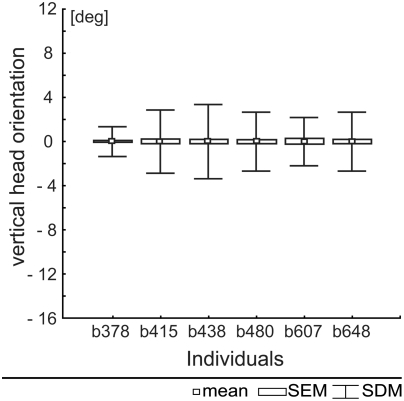
Normalised vertical head orientation angles during intersaccadic intervals in 6 birds. Vertical orientation deviations taken from 42 selected flight sequences of six individuals. The mean vertical beak orientation is zero due to normalisation. The small squares indicate mean values and boxes indicate standard error of the mean while whiskers indicate standard deviations.

Constant translational flow can only be obtained if there is also no rotational motion component around other axes of the head either. Examination of changes in pitch angle was possible only for a restricted set of our data (see above), i.e., during those sections of the intersaccadic intervals during which the bird flew parallel to the cage's frontal edge. The measurements (fig. 8) confirm the impression already obtained from visual inspection of the raw data (fig. 4). The pitch angle of the head is kept quite constant with standard deviations amounting maximally to 3.5 degrees.

## Discussion

Optic flow is an important visual source that provides information about a complex three dimensional environment. Only translational optic flow provides information about the three dimensional structure of the environment while rotational components do not [4]. It is a known strategy of insects to behaviourally separate translational from rotational components. This facilitates extraction of spatial information from behaviourally generated optic flow. Flies perform fast body saccades which are supplemented by even faster head saccades, and look in a constant direction between saccades [7]–[9], [22]. We want to compare this behaviour to avian flight.

First of all, we did not observe saccadic fast body turns as was shown for the blowfly. The force that has to be overcome when changing direction is proportional to mass and velocity. Zebra finches have approximately ten times the length of a blowfly (12 cm) and 100–140 times the mass (10–14 g). The velocities of the zebra finches in our experiment reached up to 3.5 m/s while Schilstra and van Hateren [8] measured flight velocities of only up to 1.2 m/s for the blowfly. So while in flies a significant proportion of the gaze shift is done by body saccades, such behaviour is probably impossible for zebra finches due to inertia.

However, while the body moves smoothly the head either turns rapidly or is held constant in orientation even when manoeuvring (fig. 4). We use head orientation as a first approximation for estimating gaze direction. But in contrast to flies, birds have movable eyes which we assume to contribute to gaze shifts. A recent study (Voss and Bischof, submitted) demonstrated substantial eye movements in the zebra finch. Gioanni [13]–[14] showed that during horizontal optokinetic reflexes induced by a rotating drum, eye movements are synchronised with head movements. The eye movements accounted for up to 20% of the gaze shift in that study. Thus we presume that in the zebra finch eye movements add to saccadic head shifts to optimize the saccadic gaze shift. This would be analogue to the blowfly that executes head movements to add speed and accuracy to the gaze shift generated by the body saccade [9], [22]. Between saccades, eye movement compensates for slow head movement to keep the gaze direction fixed. However, eye movement could not be resolved in our study.

The gaze shifts of birds and flies are similar not only to the fact that there are phases of fast and slow head turns, but also to some parameter values of these phases. For example, the maximum angular velocity of saccades measured by Schilstra and van Hateren [8] was about 2000°/s and for the zebra finch the fastest saccade we found was at 2150°/s. Also, rotational velocities of the gaze during intersaccadic intervals in the blowfly were found to be below 100–200°/s [9]. In birds we found a mean velocity of 115°/s during intersaccadic intervals, which presumably may further be reduced by compensatory eye movements. Schilstra and van Hateren state that these residual rotational velocities are slow enough to make motion blur from rotational optic flow negligible. They also state that during the shortest saccades, the visual system experienced significant rotational motion blur for only 15–20 ms. Due to our search parameters we did not measure saccades shorter than 8 ms, and we only called a turn a saccade when the head moved faster than 400°/s. So saccade durations given in this study always coincide with the experience of rotational motion blur. These saccade durations were 15.6 ms in mean.

We wanted to compare two morphs of zebra finches, because investigating optic flow processing in a deviating visual system such as that of the white morph might reveal some additional insight. Surprisingly, wild type and white zebra finches did not show significant differences. The strong deviations of the visual system in the white morph which were assumed to have some major influence on the AOS and thus on optic flow processing did not seem to have any effect on the overall flight performance. This is congruent to Eckmeier and Bischof [21] who did not find differences in the optokinetic nystagmus of the two morphs elicited by rotational optic flow.

Taken together, our experiments demonstrate that birds use a gaze strategy separating rotational and translational optic flow. This is achieved by an alternation of fast rotational head shifts and intersaccadic periods where head rotations are minimal. Eye movements probably enhance gaze shift during saccades and minimize it during intersaccadic intervals. To this end, head and eye saccades of birds appear to be analogous to body and head saccades in flies. Both, flies and birds, exhibit similar kinetic characteristics of gaze control. By exhibiting an active gaze strategy similar to that of the blowfly, zebra finches are able to use optic flow for distance estimation.

## Supporting Information

Video S1 The video shows one of the analysed flights. The view of both cameras are presented simultaneously. The white morph performs a left curve around the obstacle. When red circles appear the bird is going to shift gaze in a saccade.
(0.76 MB MOV)
